# Risk of dengue, Zika, and chikungunya transmission in the metropolitan area of Cucuta, Colombia: cross-sectional analysis, baseline for a cluster-randomised controlled trial of a novel vector tool for water containers

**DOI:** 10.1186/s12889-023-15893-4

**Published:** 2023-05-30

**Authors:** Maria Angelica Carrillo, Rocio Cardenas, Johanna Yañez, Max Petzold, Axel Kroeger

**Affiliations:** 1 Centre for Medicine and Society, Master Programme Global Urban Health, Albert-Ludwigs University Freiburg, Freiburg in Breisgau, Germany; 2 Vector Control Programme, Instituto Departamental de Salud Norte de Santander, Cucuta, Colombia; 3 Institute of Public Health, Gothenburg University, Göteborg, Sweden

**Keywords:** Dengue, Zika, Chikungunya, *Aedes aegypti*

## Abstract

**Background:**

Arbovirus diseases such as dengue, Zika, and chikungunya are a public health threat in tropical and subtropical areas. In the absence of a vaccine or specific treatment, vector management (in this case the control of the primary vector *Aedes aegypti)* is the best practice to prevent the three diseases. A good understanding of vector behaviour, ecology, human mobility and water use can help design effective vector control programmes. This study collected baseline information on these factors for identifying the arbovirus transmission risk and assessed the requirements for a large intervention trial in Colombia.

**Methods:**

Baseline surveys were conducted in 5,997 households, randomly selected from 24 clusters (neighbourhoods with on average 2000 houses and 250 households inspected) in the metropolitan area of Cucuta, Colombia. The study established population characteristics including water management and mobility as well as larval-pupal indices which were estimated and compared in all clusters. Additionally, the study estimated disease incidence from two sources: self-reported dengue cases in the household survey and cases notified by the national surveillance system.

**Results:**

In all 24 study clusters similar social and demographic characteristics were found but the entomological indicators and estimated disease incidence rates varied. The entomological indicators showed a high vector infestation: House Index = 25.1%, Container Index = 12.3% and Breteau Index = 29.6. Pupae per person Index (PPI) as an indicator of the transmission risk showed a large range from 0.22 to 2.04 indicating a high transmission risk in most clusters. The concrete ground tanks for laundry –mostly outdoors and uncovered- were the containers with the highest production of *Ae. aegypti* as 86.3% of all 17,613 pupae were identified in these containers. Also, the annual incidence of dengue was high: 841.6 self-reported cases per 100,000 inhabitants and the dengue incidence notified by the National surveillance system was 1,013.4 cases per 100,000 in 2019. Only 2.2% of the households used container water for drinking. 40.3% of the study population travelled during the day (when *Aedes* mosquitoes bite) outside their clusters.

**Conclusions:**

The production of *Ae. aegypti* mosquitoes occurred almost exclusively in concrete ground tanks for laundry (lavadero), the primary intervention target. The baseline study provides necessary evidence for the design and implementation of a cluster randomized intervention trial in Colombia.

**Supplementary Information:**

The online version contains supplementary material available at 10.1186/s12889-023-15893-4.

## Introduction

Arboviral diseases such as dengue, Zika and chikungunya (DZC) are a public health threat in tropical and sub-tropical countries due to the increase of the global burden promoted by the rapid spread of their mosquito vectors, *Aedes aegypti* (primary vector) and *Aedes albopictus* (secondary vector) [1, 2]. In Latin America, these diseases are mainly transmitted by *Ae. aegypti* [3] which breeds particularly in artificial water containers in close proximity to human dwellings [4]. Environmental changes, unplanned urbanization, inadequate sanitization and human activities involving water storage and deficient trash management contribute to the production of this vector [5–7]. Certain socio-demographic characteristics such as level of education [8], gender inequality [9] and crowded living conditions [10] add to the risk of *Ae. aegypti* vector infestation.

In Colombia, *Ae. aegypti* is widespread in mainly urban areas below 2300 m above the see level [11]. Suitable habitats favoured by numerous water containers and climate change have caused the proliferation of *Ae. aegypti* throughout the country [12]. The presence of *Ae. aegypti* is reflected by the high burden of dengue, Zika and chikungunya in the last years (2014–2019), 504,414 dengue cases was reported [13]. Colombia is now hyper-endemic for the dengue virus (DENV) with the circulation of all four serotypes and outbreaks occurring every 3–4 years [14]. Chikungunya and Zika were detected for the first time in the country in 2014 and 2015, respectively [15, 16], and since their introduction 488,378 cases of chikungunya and 109,995 cases of Zika have been reported until 2019 [11, 17–21]. Moreover, Zika has been associated with the development of several neurological complications including microcephaly and Guillain–Barre syndrome [22]. In the absence of antiviral treatment for arboviral infections and of a dengue vaccine suitable for public health use, the key measure for preventing the transmission of DZC continues to be vector control. Effective vector control depends on a sound understanding of larval and adult vector ecology of which little is known in hyperendemic cities of Colombia.

The transmission risk of arboviral disease outbreaks in endemic regions has traditionally been assessed by larval indices (*Stegomyia* indices) [23–25], complemented or replaced by pupal indices [26] as pupae are a reliable proxy for adult mosquito abundance [27, 28]. Given the suitability of domestic breeding habitats for *Ae. aegypti*, mosquito management within households is crucial to the control of DZC. Moreover, understanding factors that drive abundance and persistence of *Ae. aegypti* in the household environment will contribute valuable information to the design and implementation of efficient and targeted vector control strategies. In Cucuta, a major city in Colombia, where the average annual dengue incidence is 668 per 100.000 persons (it is twice as high as the national average with 282 cases per 100 000) [29], little evidence is known on vector infestation level and its risk factors.

This study describes socio demographic characteristics, identifies breeding habitats and establishes container productivity profiles and level of infestation *of Ae. aegypti* and provides an epidemiological profile. The purpose of this study is to collect baseline information for a cluster-randomised trial (CRTs) that seeks to reduce DCZ risk in Colombia through the implementation of new novel vector control method (insecticidal coating for domestic containers). CRTs are a robust design for measuring the effect of interventions conducted at community level [30]. Several CRTs have shown an impact on vector densities [31–34], but few studies provided evidence of the impact on dengue incidence. The Camino Verde trial, which assessed the impact of community mobilization on dengue incidence, has been a quite unique example [35]. The development of novel approaches to vector control requires well-designed field trials with a larger number and size of cluster and/or longer study periods [36, 37]. With the main goal of reducing the burden of arbovirus diseases, this study was designed with a large and sufficient number of clusters to assess the effectiveness of insecticidal coating in key domestic water containers for reducing the transmission of DENV, CHIKV, and ZIKV. Findings from the here presented baseline study will show the complexity of preparing a large trial and provide baseline information about different factor influencing the transmission of arbovirus diseases.

## Methods

### Place of study

The study was conducted within 2.5 months in 2019–20 in Metropolitan Cucuta in the North-East of Colombia, including the city of Cucuta (629,414), and two adjacent municipalities Villa del Rosario (93,735) and Los Patios (81,411 inhabitants) located in the Norte de Santander state [38]. The climate is warm and dry, with a mean annual temperature of 26.7 °C and annual rainfall of 806 mm. There are two rainy seasons from the end of March to the beginning of June and from the end of September to the beginning of December. DZC are endemic, with occasional epidemic outbreaks [39].

### Study design and sample size

This is the baseline study (using a cluster design) for a large cluster randomized trial on the impact of treating water containers with a protective paint. The sample size was calculated for detection of a 50% reduction in the House Index with > 99% power at 5% significance level. Given a baseline HI of 30%, an intra-cluster correlation coefficient (ICC) of 0.01 and a cluster size of 2,000 households it was found that a minimum of 12 clusters per study arm was needed [40]. The same result but with a lower power (68%) was found for a baseline DZC incidence of 3% and a 50% reduction in the intervention arm. A large number of households per cluster was needed as *Ae. aegypti* vectors are day-biters [41–43] and it was assumed that many household members stay within their neighbourhoods during the day (mainly housewives, small children and school children when the school is close to their house) where they are at risk to get infected. All estimations were done through The Shiny CRT Calculator which is a web-based app to determine sample size and power for cluster trials [44].

### Sampling procedure for household survey and entomological inspections

**Fig. 1:**
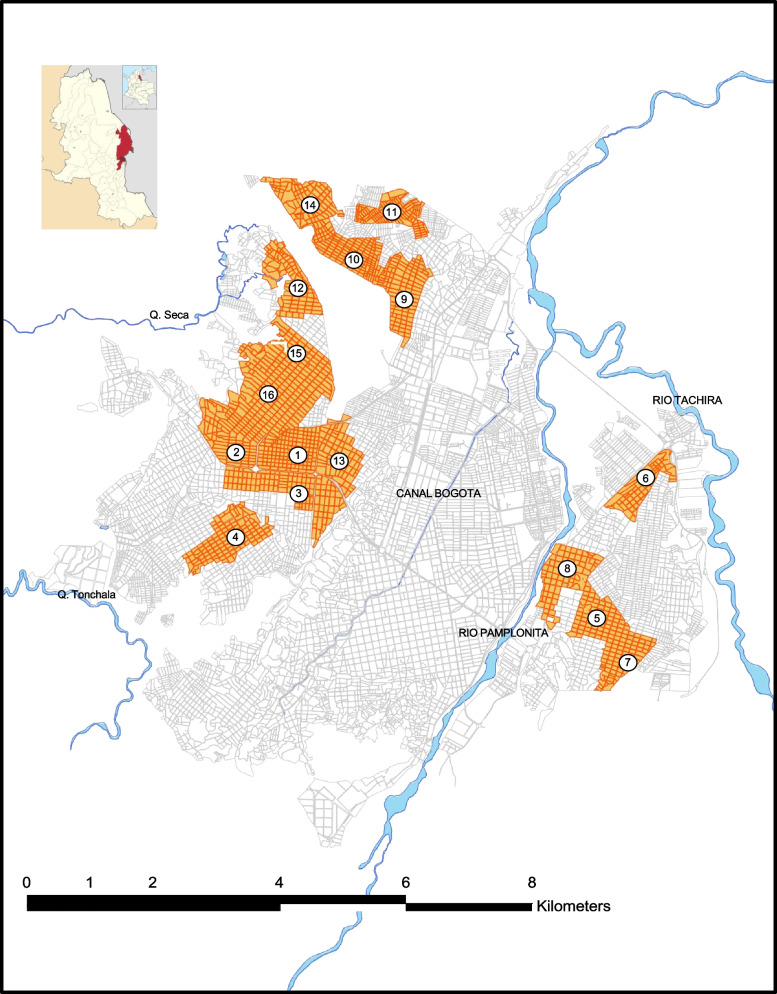
Selection of clusters in Cucuta Map shows the 16 clusters selected in Cucuta which were visited by interviewers and vector control staff

On the basis of available maps, the city was stratified into high, middle and low endemic/infested areas for *Ae. aegypti* (strata) using the surveillance information from SIVIGILA of the preceding year as well as information obtained from interviews with the vector control coordinators in Cúcuta. By this way, 24 high and medium endemic areas (clusters) were selected in Cucuta (16 clusters), Los patios (4 clusters) and Villa del Rosario (4 clusters). Figure 1 shows the selected clusters in Cucuta to be sampled by staff. For the baseline study they were not yet paired and allocated to the intervention and control arm as this was planned to do with the information resulting from the baseline study enabling us to find pairs of clusters with similar characteristics. The following characteristic should be similar in each pair of clusters: (I) High and middle level endemicity for *Aedes*-borne diseases; (II) Similar socio-economic conditions; (III) Similar types of the built environment: houses, schools, industry, health centers, workshops, churches and stores; (IV) Similar characteristics of public spaces. Within each cluster, all blocks of houses were inspected including public spaces. For the entomological/socio-demographic household survey, a proportion of 10% of 2000 houses was sampled in this study, resulting in 200 houses in each cluster. This number was rounded to 250 by convenience. Therefore, the study targeted 6000 households (250 households in each cluster) using a systematic random sampling method. For this we first estimated the sampling interval by dividing the total number of households in each cluster (around 2000) by the number of sample households (250), resulting in a sampling interval of 8. All households located within each cluster were mapped (using Google Maps) and a randomly defined starting point was selected and from there every 8th household was visited. Clear replacement rules were given to the interviewers and the compliance was checked in sample of families.

Vector control staff and trained interviewers participated in the survey. They were divided into “couples” of one vector inspector and one interviewer. Each couple was assigned one cluster where they had to visit the sample houses. The interviewer did the interview with the head of household and simultaneously the inspector (local vector control staff) did the inspection of water containers both of them filling a form.

### Data collection

The baseline surveys included a household questionnaire and entomological survey (see Additional files 1 and 2). The instruments were discussed in a participatory workshop with experts and pre-tested among the members of vector control staff in the three municipalities leading to some modifications. The first instrument was the questionnaire which was applied by trained interviewers and the other part was the form for the entomological inspections (larvae/pupae survey) which was applied by the vector control staff. As this is part of the national vector control programme only verbal consent of the house owner was obtained to check their water containers. An extensive training was conducted by the research team before starting the field work.

### Household questionnaire

Demographic and health data was collected through face-to-face interviews using a standard questionnaire that included both structured and semi- structured questions. Household questionnaire was adapted from published research in Colombia [45]. The questions referred to socio-demographic parameters (number of people living in the house, age, sex, and educational level), self-reported DZC disease acquired during the last months, population mobility during the day, usage of water in tanks and willingness to accept the intervention trial (see Additional file 1).

### Entomological survey

The standard entomological survey form was adapted following the guidelines of Standard Operational Procedures (SOPs) by WHO [46]. The questions were adapted to local conditions and survey requirements (see Additional file 2). The following data were recorded from each household: total number of containers (potential breeding sites), number of mosquito larvae positive and negative containers (any species), pupae count per container, container type, and other container characteristics (if they were covered or uncovered, outdoors or indoors).

### Dengue surveillance and case definition

Data of the national surveillance system were obtained from SIVIGILA (the national health surveillance system), aggregated by year and setting (study areas) over the study period. Notified dengue cases including those classified as dengue fever (DF) and severe dengue, relying on a clinical case definition or lab confirmed or hospitalized patients. This study used annual population data (from the National Institute of Statistics-DANE; [47]) for calculating the incidence rate.

### Data management and analysis

Descriptive analyse and double data entry was practiced (to minimize data entry errors) into a database using Microsoft Office Excel software by an assistant and supervised by the research team. The analysis was done using SPSS software version 28.0.1.1 (15).

Socio-demographic data were entered in a database. We assessed the frequency of variables potentially associated with the outcomes of recent dengue virus infection (sex, age, educational level, peoples` mobility) and self-reported dengue fever in household members. Persons’ Chi-square test (χ2) was applied to determine the differences between population characteristics and DZC self-reported cases, and peoples` mobility across all settings. All statistical analyses were performed at a 0.05 significance level.

Entomological indices were analysed per cluster and overall to show the presence, distribution and abundance of *Ae. aegypti* and the breeding sites most productive for adult mosquitoes were identified [48].

House index (HI): Percentage of houses infested with larvae and/or pupae.

Container index (CI): Percentage of water-holding containers infested with larvae or pupae.

Breteau index (BI): Number of positive containers per 100 houses inspected.

Pupae per person (PPI): Number of pupae per person in each household.

To estimate the pupal count for large container (more than 20 L) the methodology by Romero-Vivas [49] was used: According to water level, the number of pupae found was multiplied by a calibration factor.

### Ethical considerations

The household questionnaire was only applied to adults who provided information related to the purpose of this study. No child or adolescent below the age of 18 was interviewed in this study. All people participating in the study were informed in local language through the study information sheet in a written and oral way. They were asked to sign the informed consent form. All participants were informed that their participation was voluntary and that their responses remained anonymous, therefore the study used numbers which replaced the names of individuals and codes which replaced the address of house. Before examining the domestic and peri-domestic water-holding containers, the field team requested permission to enter the house, did the inspection and collected entomological and sociodemographic data.

The study received approval from local health authorities in Cucuta and Norte de Santander and the study protocol was approved by the ethical committee of the Albert–Ludwigs-Universität (application number 141/19) in Freiburg, Germany and the National Institute of Health in Bogota, Colombia.

## Results

In the baseline survey, a total of 5,997 households with 23,408 people in 24 clusters were visited by the field team. Some data were missing, or some questions were not answered by household participants, these values were excluded from the analysis.

### Socio-demographic information

Of the household members 53.3% (12,448/23,357) were women (Table 1). The mean number of people living in a household was 3.9 (range 1–12, SD = 1.9). The mean age of the study population was 35 years (range: 2 months-105 years, SD = 21.7). The largest age group was between 35 and 64 years (35.8%; 8,375/23,365). Almost half of the study population above 5 years of age (44.9%; 10,240/22,817) reached secondary education and a significant proportion (31.9%) reached primary education. (The educational level was analysed for 5 years and older as the primary school starts at the age of 5 years in Colombia [50]). Similar characteristics were found in each cluster. (A complete description of all clusters can be found in the Additional file 3).

**Table 1:** Population characteristics and self-reported annual incidence of DZC

Characteristics	Variable	N*	Proportion %	Frequency of DZC during last 12 months % (n/N)**	P***
Sex	Female	12,448	53.3%	2.3% (285/12,448)	0.001
	Male	10,909	46.7%	1.7% (183/10,909)	
	Total (excluding 51 missing values)	23,357			
Age groups	1 < year	122	0.5%	0.8% (1/122)	0.001
	1–4 years	1,172	5.0%	2.5% (29/1,172)	
	5–9 years	1,608	6.9%	3.7% (6071,608)	
	10–19 years	3,849	16.5%	1.9% (73/3,849)	
	20–34 years	5,677	24.3%	1.7% (99/5,677)	
	35–64 years	8,375	35.8%	1.8% (147/8,375)	
	> 64 years	2,562	11.0%	2.3% (59/2,562)	
	Total (excluding 43 missing values)	23,365			
Educational level	No education	745	3.5%	2.1% (16/745)	0.423
	Primary education	7,215	33.5%	2.1% (152/7,215)	
	Secondary education	10,251	47.6%	1.8% (187/10,251)	
	Higher education	3,316	15.4%	2.2% (73/3,316)	
	Total (excluding 587 missing values and 1,294 children under 5 years)	21,527			
Mobility during the day	Inside cluster	13,103	59.7%	2.2% (291/13,103)	0.03
	Outside cluster	8,842	40.3%	1.7% (146/8,842)	
	Total (excluding 1,463 missing values)	21,945			

^*^N number of people responding excluding missing values; **n number of people who reported to have had dengue divided by the total number of people who responded. ***p-values based on Pearson's chi-squared test

### Peoples’ mobility

The insect vector *Ae. aegypti* bites primarily during the day inside or around houses and has a limited flight range; therefore, people’ mobility during the day is important to know when assessing the place of dengue transmission [51]. High mobility will make it less likely to show a protective effect by household interventions in a CRT. Therefore, we decided for a large cluster size where the majority of inhabitants move inside clusters during the day. We found in the household survey that the majority of the study population (59.7%; 13,103/21,945) remains inside the cluster boundaries while a considerable proportion, 40.3% (8,842/21,945) moves to places outside their cluster where they are exposed to vector bites. A Pearson's chi-squared test was performed to compare the proportion of daytime spent outside cluster by each member of household in different population characteristics, which showed there was a statistically significant difference among sex (X^2^ = 300.27, df = 1, P = 0.001), the age groups (X^2^ = 1593.58, df = 5, *P* = 0.000) and the education level of population (X^2^ = 1575.28, df = 3, *P* = 0.000). Those who moved outside their cluster were mainly male (53.8%; 4753/8836), young adults and adults between 20 to 64 years old (72.4%; 6,398/8,823) and people in secondary education (50.7%; 4,329/8,537).

### Epidemiological information

**Fig. 2:**
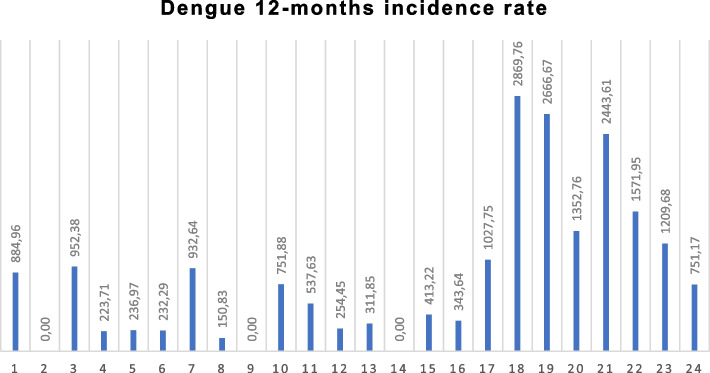
Self-reported dengue per cluster in the previous 12 months The bar graph shows 12-months incidence rates per 100,000 inhabitants. Cúcuta: cluster 1–16; Los Patios: cluster 17–20; Villa del Rosario: cluster 21–24

Across all clusters, 2.0% (468/23,093) of household members reported to have had any arboviral disease (DZC) during the last 12 months (Dengue incidence was 0.84%). This ranged from 3.0% (116/3,814) in Los Patios, 2.3% (90/3,948) in Villa del Rosario, to 1.7% (262/15,331) in Cucuta. A small group of respondents (68 persons) assumed to have had mixed infections with two arbovirus diseases. The 12-months self-reported incidence was the following: dengue 841.6 per 100,000 inhabitants, Zika 585.3 per 100,000 inhabitants and chikungunya 572.45 per 100,000 inhabitants. Persons with dengue (83.2%) usually went for diagnosis and treatment to the hospital while persons with Zika or Chikungunya used much less the hospital services (45.3% and 57.5% respectively). Figure 2 shows the reported Dengue incidence rate by study population in 24 clusters. Los Patios (1,965.9 per 100,000 inhabitants) and Villa del Rosario (1,494.4 per 100,000) noticeably had a higher dengue incidence rate than Cucuta (402.9 per 100,000). Cluster 18 had the highest dengue incidence rate (2,869.8 per 100,000 inhabitants) and clusters 2, 9 and 14 did not report any dengue case (Fig. 2).

Table 1 shows the annual incidence of self-reported DZC related to sociodemographic characteristics (statistically significant differences except for education level). Females had a higher incidence (2.3%; 285/12,448) than males (1.7%; 185/10,909) (X^2^ = 11.09, df = 1, *P* = 0.001). Younger age groups, particularly those between 5 and 9 years of age (3.7%; 60/1,608) reported more cases of arbovirus diseases in comparison with other age groups (X^2^ = 32.66, df = 6, *P* = 0.001). Educational level (as an indicator for socioeconomic characeristics) was not associated with disease incidence. People who stayed inside their clusters during the day had a higher incidence (2.2%; 291/13,103) than those who move during daytime outside their home DZC (1.7%, 146/8,842; X^2^ = 8.778, df = 1, *P* = 0.03).

### Dengue incidence notified by the National surveillance system, 2015–2021

During the 7-year period, 2015–2021, 8,190 dengue cases were notified by SIVIGILA in the study areas. The number of annual case notifications varied from a low of 333 cases in 2020 (173.1 per 100,000 population) to a high of 1,949 cases in 2019 (1,013.4 per 100,000 population), with a mean of 1,170 cases per year (incidence 608.4 per 100,000 population). Across all clusters, the number of dengue cases varied with a range of 4 to 193 dengue cases between 2015–2021. Cluster 14 had the lowest dengue incidence rate (51.1 per 100,000 population) in 2020 and Cluster 5 had the highest incidence rate (2,933.8 per population (see Table 2). Patients with fever and other DZC symptoms resort to the health services only when they feel severely sick.

**Table 2:** Annual dengue incidence per 100,000 people notified by SIVIGILA

Cluster	2015	2016	2017	2018	2019	2020	2021
1	870.7	245.3	220.8	686.8	1,410.4	245.3	1,802.8
2	607.5	341.7	88.6	734.1	1,151.8	139.2	1,316.3
3	564.3	435.3	322.5	645.0	1,015.8	225.7	854.6
4	557.1	330.8	243.7	1,532.0	1,410.2	139.3	1,009.7
5	1,420.1	764.7	577.4	1,201.6	2,933.8	577.4	811.5
6	776.8	263.1	263.1	839.5	814.4	137.8	451.1
7	298.5	155.2	107.5	418.0	835.9	107.5	238.8
8	981.7	387.5	374.6	632.9	1,433.7	439.2	426.2
9	608.6	294.5	215.9	981.5	795.1	157.0	647.8
10	501.6	178.4	156.1	802.6	412.4	78.0	646.5
11	520.8	186.7	147.4	668.2	599.4	78.6	343.9
12	824.8	477.5	130.2	1,128.7	803.1	238.8	2,452.8
13	905.6	235.7	285.3	372.2	2,009.7	173.7	831.2
14	664.5	127.8	115.0	805.0	396.1	51.1	907.2
15	583.3	272.2	108.9	840.0	770.0	147.8	754.5
16	990.2	452.1	301.4	1,819.0	2,077.3	236.8	1,410.0
17	301.2	283.4	301.2	513.7	974.3	230.3	885.7
18	331.0	296.8	159.8	993.0	707.7	57.1	764.8
19	224.4	366.2	248.1	826.8	1,169.4	141.7	685.1
20	555.9	465.4	245.6	1,745.3	736.9	116.4	452.5
21	1,395.8	197.2	227.6	1,395.8	880.0	197.2	1,213.8
22	1,252.1	445.5	337.1	1,131.7	517.7	96.3	565.9
23	496.6	264.9	242.8	1,390.6	640.1	121.4	684.3
24	605.1	357.5	288.8	1,883.9	440.0	233.8	1,265.1
**Overall**	**688.4**	**316.1**	**230.3**	**989.0**	**1,013.4**	**173.1**	**848.6**

Table shows the annual dengue incidence from 2015 to 2021 in all study clusters

### Entomological information

Entomological inspections were conducted in all 5,997 households using the Standard Operational Procedures (SOPs) of the vector control services. Table 3 presents the larval and pupal indices in the 24 clusters from the three settings. The overall house index (HI) was 25.1% (1,504/5,997), the container index (CI) was 12.3% (1,776/14,386) and the Breteau index (BI) 29.6 per 100 houses (1,776/5,997) which shows high vector infestation and above the 5% assumed by WHO with the potential of epidemic disease transmission [52]. The HI was significantly different among the different clusters (σ2 = 0.0081, SD = 0.09, range = 6%-37.6%). The CIs were generally above 3% except for cluster 22 (2.25%). The BI values were highest in cluster 23 (48.8; 122/250), cluster 12 (46.8; 118/252) and cluster 24 (45.2; 113/250) and the lowest in cluster 18 (6.8; 17/250). The Pupae per person index (PPI) varied among clusters, with a range from 0.22 to 2.04 (SD = 0.49, σ2 = 0.2404), most PPI values being between 0.5 and 1.5, indicating a considerable risk of epidemic transmission [53].

**Table 3:** Vector infestation in study clusters

Cluster	Houses	Persons	deposits	Positive houses	Positive deposits	Pupae count	HI	CI	BI	PPI
**Cucuta**	**3,997**	**15,645**	**8,541**	**1,048**	**1,240**	**13,800.4**	**26.2%**	**14.5%**	**31.02**	**0.88**
1	280	1,130	600	94	102	1,383.7	33.6%	17.0%	36.43	1.22
2	212	905	426	41	47	601.2	19.3%	11.0%	22.17	0.66
3	265	1,050	557	72	82	504.5	27.2%	14.7%	30.94	0.48
4	235	894	358	45	49	192.9	19.1%	13.7%	20.85	0.22
5	230	844	554	81	94	985.9	35.2%	17.0%	40.87	1.17
6	250	861	743	72	76	582.1	28.8%	10.2%	30.40	0.68
7	272	965	607	42	43	960.5	15.4%	7.1%	15.81	1.00
8	247	663	563	88	105	1,150.2	35.6%	18.7%	42.51	1.73
9	265	1,066	342	87	95	1,074.5	32.8%	27.8%	35.85	1.01
10	267	1,064	358	50	57	1,446.7	18.7%	15.9%	21.35	1.36
11	234	930	308	63	83	281.2	26.9%	26.9%	35.47	0.30
12	252	1,179	703	82	118	290.8	32.5%	16.8%	46.83	0.25
13	223	962	737	60	92	1,238.3	26.9%	12.5%	41.26	1.29
14	235	1,000	313	80	89	696.7	34.0%	28.4%	37.87	0.70
15	250	968	735	37	49	724.5	14.8%	6.7%	19.60	0.75
16	280	1,164	637	54	59	1,686.7	19.3%	9.3%	21.07	1.45
**Los Patios**	**1000**	**3,815**	**2,525**	**133**	**142**	**2,327.8**	**13.3%**	**5.6%**	**14.20**	**0.61**
17	250	973	783	38	40	549.8	15.2%	5.1%	16.00	0.57
18	250	906	754	15	17	580	6.0%	2.3%	6.80	0.64
19	250	975	591	53	55	774.5	21.2%	9.3%	22.00	0.79
20	250	961	397	27	30	423.5	10.8%	7.6%	12.00	0.44
**Villa del Rosario**	**1000**	**3,948**	**3,320**	**323**	**394**	**4,272.4**	**32.3%**	**11.9%**	**39.40**	**1.08**
21	250	1,064	643	58	64	588.9	23.2%	10.0%	25.60	0.55
22	250	827	751	77	95	1,686.1	30.8%	12.6%	38.00	2.04
23	250	992	1,088	94	122	1,498.9	37.6%	11.2%	48.80	1.51
24	250	1,065	838	94	113	498.5	37.6%	13.5%	45.20	0.47
**Total**	**5,997**	**23,408**	**14,386**	**1,504**	**1,776**	**20,400.6**	**25.1%**	**12.3%**	**29.61**	**0.87**

*HI* House index, *CI* Container index, *BI* Breteau index, *PPI* Pupae per person index

### Most infested container types

**Fig. 3:**
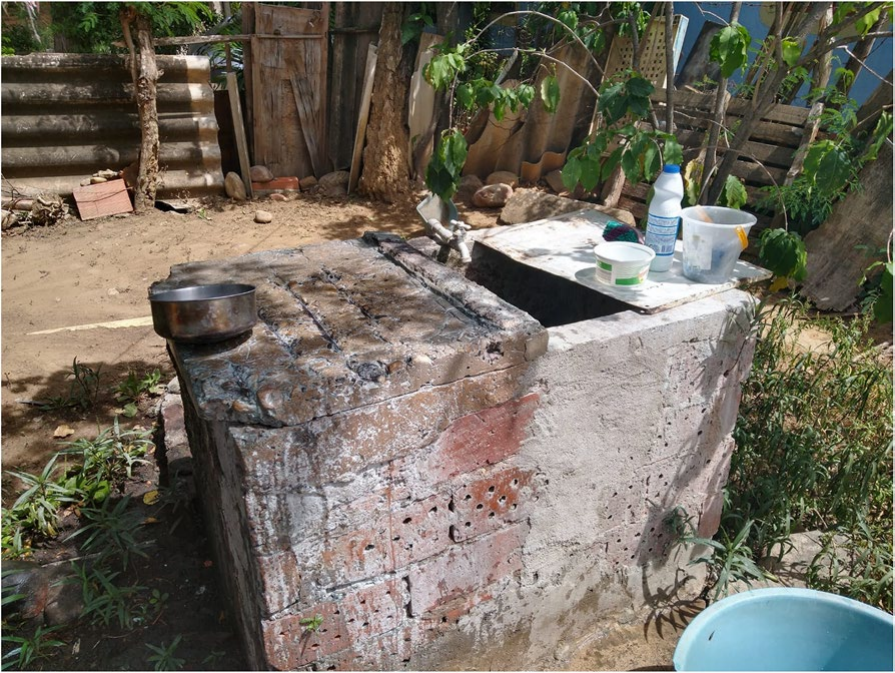
A concrete ground tank for washing purpose Concrete ground tanks for washing (*lavadero*) are made of cement or ceramic tiles with the shape of a rectangle or square and variable sizes (size measurement in the photo)

A total of 14,386 water holding containers were found in the entomological inspection. These included containers used for water storage as well as some discarded mainly small containers not used for water storage but in which water had accumulated. Overall, the most common containers were concrete ground tanks (48.5%; 6,975/14,386) of which concrete tanks for washing purposes (79.3%; 5,528/6,975) were the most common ones (Fig. 3). Other container types included elevated tanks (24.5%; 3,529/14,386), plastic tanks (11.3%; 3,529/14,386) and buckets (10.1%; 1,446/14,386). Among the positive containers, the most infested water containers with *Ae. aegypti* larvae were ground tanks for cleaning and washing purposes (71.9%, 1277/14,386). Table 4 shows the distribution of water containers. The distribution of containers was similar in all clusters. (A description of the distribution of containers per cluster and setting can be found in the Additional file 3).

**Table 4:** Types of water containers inspected and pupae productivity

Type of containers	# containers	%	Positive container	%	Pupae count (n*)	%* Productivity
Concrete ground laundry tank (*lavadero*)	5,528	38.4%	1,277	23.1%	17,613.2	86.3%
Concrete ground tank for water storing	1,447	10.1%	119	8.2%	1,661.1	8.1%
Plastic tank	1,624	11.3%	119	7.3%	688.8	3.4%
Buckets	1,446	10.1%	138	9.5%	238	1.2%
Elevated tank	3,529	24.5%	30	0.9%	124	0.6%
Metal barrel	137	1.0%	30	21.9%	46.5	0.2%
Sinks	67	0.5%	6	9.0%	8	0.0%
Flowerpot	95	0.7%	21	22.1%	8	0.0%
Others (including natural habits)	72	0.5%	9	14.1%	5	0.0%
Jars/pots	118	0.8%	5	4.2%	4	0.0%
Wheels	75	0.5%	15	20.0%	4	0.0%
Fuel jars (pimpinas)	121	0.8%	3	2.5%	0	0.0%
Water bottles	127	0.9%	4	3.1%	0	0.0%
Total	14,386		1,776	12.3%	20,400.6	

Pupal productivity: n* = total number of pupae for all containers of that type; %* = (total number of pupae from that container type/overall total number of pupae) × 100

### Pupal productivity in different water container types

The total number of pupae collected across all cluster was 20,400. Of these 13,800 pupae were collected from the clusters in Cucuta (271 households) with a median of 50.9 total pupae per household; 2,328 pupae were collected from the 4 clusters of the municipality of Los Patios (80 households) with a median of 29.1 and 4,272 from the 4 clusters in the municipality of Villa del Rosario (190 households) with a median of 22.5 pupae per household.

The water container type with the highest proportion of pupae was the concrete ground tank, producing 94.5% of all pupae as a proxy for the production of adult mosquitoes (Table 4). From these, tanks for washing and cleaning (*lavaderos*) were noticeably the most productive water containers for *Ae. aegypti* (86.3%). This is probably related to the fact that most of these ground tanks were uncovered and outside of the house. Less important for pupal production were plastic tanks, producing 3.4% of pupae and least important were buckets, elevated tanks and metal barrels, producing together only 2.0% of pupae (Table 4).

### Ground tanks and water use

Characteristics of the concrete ground tanks (*lavaderos)* were similar in all clusters. Most of them were mainly used for washing and cleaning, without a lid (92.5%; 5,017/5,421) and located outdoors (60.1%; 3,259/5,421). The mean number of ground tanks was 1 per household (SD = 0.41, range = 0–4). Overall, the mean capacity of these containers was 363 L (range 20 L to 5,000 L). Traditionally, these ground tanks have been treated by house owners with another no-standardized vector control method, chlorine tablets, which are self-applied by a member of household. Almost half of participant households (46.4%) reported ever having applied chlorine tablets*,* however the doses and frequency of application was variable. A small proportion (6.8%) of the population reported to use fish as biological control in these containers.

Regarding the sources of drinking water, only 2.2% (133/5,997) of the houses take drinking water from these ground tanks while the majority uses it for laundry (97.8%) and drink water from the tap provided by the municipal water company. We observed in our survey that people had quite a strict separation of the water sources for drinking and cleaning or washing (water mostly from ground tanks or plastic tanks). Drinking water also is used for cooking, showering and flush toilets. 

### Willingness to receive a new vector control intervention

The community in all clusters was interested in receiving the proposed novel vector control method (insecticidal coating for water containers). Overall, 88.3% of respondents held this view and there was no significant difference between clusters (5,296/5,997). The experience of the vector control staff (24 inspectors) was assessed through a short questionnaire; they considered that this study was very useful and interesting for improving public health in the city particularly regarding vector borne diseases, but they wanted to receive more training on arbovirus diseases and all of them would like to participate in the next phase of the study.

## Discussion

This study is designed as the baseline study for a large Cluster Randomized Trial (CRT) but provides also by itself important information on the: epidemiological, entomological, and socio-demographic characteristics and assesses the transmission risk of arboviral diseases in an endemic area of Colombia. 

### Vector infestation level

The field team reported similar socio demographic characteristics in all clusters, but entomological characteristics were varied. High vector densities were found throughout all clusters. All clusters had a HI above 5% (range 6.0%-37.5%; Table 3) which is sometimes assumed to be a rough proxy measure of the risk threshold of epidemic transmission. Most clusters had a CI and BI above 5% and 20, respectively [52], similar to other Latin American countries, [8, 54]. Furthermore, the pupae per person index (PPI) as a proxy measure for *Ae. aegypti* densities was high in most study clusters (range: 0.22 and 2.04) with 18 clusters out of 24 being above 0.5 [53]. These data suggest high risk of arbovirus transmission by *Ae. aegypti* highlighting the urgent need for an effective intervention.

### Main Ae. aegypti breeding sites

Concrete ground tanks were the most common type of water containers in all clusters. The population in Cucuta has historically collected water for multiple uses as a common practice of their daily routine due to the occasional shortage of tap water which has been a continuous problem in the region and other parts of the country [8].

The highest pupae production (i.e. % of all pupae in a special container type) was found in these ground or laundry tanks (86.3%). Pupal counts provide a more precise estimate of vector abundance than larval surveys [55, 56]. Thus, the vast majority of *Ae. aegypti* mosquitoes are developing in *lavaderos*, which are filled with water for washing clothes and cleaning purposes. Similar findings were made in other parts of Colombia and Latin America [27, 57–60] where different terms were used such as *tanquilla or alberca* [61]. Our study supports the recommendation that this type of container has to be targeted for any kind of vector control intervention in those areas where it has a high pupal productivity.

### Vector control

In Colombia, vector control, such as the application of the organophosphate temephos in ground water containers, is generally only practiced in epidemic situations. For routine control, communities are recommended to keep containers covered and clean [62]. In our study, almost half of the population (46.4%) reported putting chlorine tablets (which they purchase on the street or on the market) in the water of their laundry tanks as vector control method. Chlorine has been used to clean and eliminate immature stage of mosquitoes in water containers [63, 64] but there is only scarce evidence on the use of chlorine tablets regarding its efficacy against insect larvae in laundry tanks, and there is no information on dosage and the safe use of chlorinated water for drinking.

This study showed that almost half of the population (48.7%) had not received any health education or inspection by vector control staff in their houses. The vector control program should reach more houses particularly where they have not applied any vector control method. Studies have demonstrated a significant benefit of involving adding communities and other stakeholders in the existing government DZC control programme [35, 65, 66]. The human health dimension of ecosystem health (ecohealth) has been a successful eco-bio-social approach used in vector control programs [67, 68]. The application of eco-friendly vector control tools in combination with environmental management practices and community mobilization has been proven effective in reducing infection risk in endemic countries [69]. Studies in India and Bolivia demonstrated that engaging women and community can reduce mosquito abundance and dengue risk [70]. Moreover, the use of information and communication technology (ICT) particularly mobile phones for dengue prevention has increased in the last decade, showing to be useful for introducing behavioural change [71]. These approaches can be implemented by vector control program in Colombia to enhance communication on arboviral diseases, increase acceptance in the use of new vector control tools and reach more people.

### Considerations for the forthcoming intervention study

This study provides cluster specific information on socio-economic indicators and cluster size, on the estimated mosquito abundance, the main breeding places and their pupal productivity, the type and size of water containers, water use for drinking and cleaning which should be considered for the planning of the intervention (covering the productive water containers with a protective and transparent coating) and implementation of the CRT in randomly assigned clusters (using incidence rates and entomological indicators) into intervention and control clusters. The intervention in these ground tanks should be safe for human consumption as a small proportion of households (2.2%) uses the water is also for cooking and drinking. Moreover, people’s willingness to accept the insecticidal coating in their laundry tanks was high in all clusters (88.3%) based on the information, that a sub-study with water samples (to be presented in a different paper) showed no toxicity of the paint and will be delivered for free. Previous studies in Nepal and Bangladesh found that the application of insecticidal coating on house walls was safe and well accepted by communities and health workers. High acceptance (94%) of the households’ participants was reported in the control of leishmaniasis and Chagas disease [72, 73]. Household members perceived a decrease in mosquito and sandfly presence in the house [74].

A specific challenge for assessing the effectiveness of vector control interventions is people’s mobility during the day as 40.3% of the people in our sample used to leave the cluster area during the day at least for some hours so that they can get infected in other places and bring the viruses home. Male, adult people (20 years and older) and people in secondary education had higher proportion of time spent outside their clusters. This finding is similar to a study in Mexico which found differences in human mobility according to gender and age [75]. These population groups may play an important role in arbovirus dispersion reducing the effect of household interventions.

The novel insecticidal coating contains two active ingredients pyriproxyfen and alphacypermethrin which have both been widely evaluated for vector control. The concept of coating of surfaces with micro-encapsulated insecticidal/larvicidal products for vector control has gained special attention in comparison with other vector control methods such as insecticide-impregnated bednets, and indoor residual spraying [76, 77]. Its prolonged efficacy (for 9 to 12 months) in vector control and the excellent acceptance by communities [78–80] make it a potential alternative for *Ae. aegypti* control. It is suggested that this can be also applied by communities themselves and other affordable “do-it-yourself” strategies [76]. The ability and willingness to pay for disease prevention is also reflected in a growing private sector interest to offer such products to private and commercial customers [81]. In Colombia, insecticidal products are registered by the national authority (INVIMA) and some of them have been validated and tested through efficacy and feasibility studies with encouraging results [82]. However, standardized protocols and large-scale trials are needed for evaluating cost-effectiveness of dengue reduction as we are proposing in our study.

### Limitations

Although some studies have shown an association between arbovirus infection and occupation [83, 84]. Other studies showed no clear association [85]. Information related to occupation of the head of household was not collected in this study. Many people do not like to be asked for employment and income which has been reported as a challenge in household surveys [86]. Additionally, there was no time for a longer questionnaire due to the different activities to be conducted during the household visit. However, our target population was rather homogeneous in terms of social class.

## Supplementary Information

**Additional file 1.**

**Additional file 2.**

**Additional file 3.**

## Data Availability

All data generated or analysed during this study are included in this published article (Additional file 3).
