# Supplementary material for: Risk of dengue, Zika, and chikungunya transmission in the metropolitan area of Cucuta, Colombia: cross-sectional analysis, baseline for a cluster-randomised controlled trial of a novel vector tool for water containers
Source: BMC Public Health. 2023 May 30;23:1000. doi: 10.1186/s12889-023-15893-4 (PMC10227988; doi:10.1186/s12889-023-15893-4)
Supplement: Supplementary file 1 — Additional file 1. [file 12889_2023_15893_MOESM1_ESM.docx]

Household questionnaire

Form for extraction of socio-demographic data

*We are evaluating a new strategy against dengue. If you allow me, I will ask a few short questions that will not take up much of your time. If you feel uncomfortable with any question, you cannot answer or end the interview whenever you want.*

House code: _________________________ Cluster code: ____

1. **¿** **Could you give us the following details about the people who live in your house, including yourself in the following table?**

**Part A: Household sociodemographic characteristics**

| Person | Age | Sex  1=F  2=M  3=O | Education  Primary school=1  High school= 2 University= 3 No= 4 | During this year, did you or anyone in your household have DZC?  1=Dengue 2=Zika 3=Chikunguna 4=No | Which month? | Did you go to the doctor or health unit?  1=Yes 2=No | During the day, where do you spend most of the time (7 am -5 pm)? 1=Inside neighborhood, 2=Outside neighborhood |
| --- | --- | --- | --- | --- | --- | --- | --- |
| **1** |  |  |  |  |  |  |  |
| 2 |  |  |  |  |  |  |  |
| 3 |  |  |  |  |  |  |  |
| 4 |  |  |  |  |  |  |  |
| 5 |  |  |  |  |  |  |  |
| 6 |  |  |  |  |  |  |  |
| 7 |  |  |  |  |  |  |  |
| 8 |  |  |  |  |  |  |  |
| 9 |  |  |  |  |  |  |  |
| 10 |  |  |  |  |  |  |  |
| 11 |  |  |  |  |  |  |  |
| 12 |  |  |  |  |  |  |  |

**Part B: Practices on dengue and water use**

| 1. **Do you apply some type of vector control in your laundry tank to prevent dengue?** |
| --- |
| 1= Use of fish 2 = Addition of chlorine 3= Use of tank lid 4=Washing and brushing 5= None 6=I don’t know |
|  |
| 1. **Has the health service applied any type of powder or tablet insecticide in your laundry tank this year?** |
| 1= Yes 2= No 3= I don’t know   1. **Which deposits do you use for water consumption?**   1= Ground tank 2=Tap water 3=Other deposits, specify____________ 4=None   1. **Do you know where mosquitoes lay their eggs or where they breed?**   1=Clean water 2=Dirty water (e.g. puddle) 3= Grass or pasture 4= I don’t know   1. **¿** **which of these media would you like to receive information about dengue?** *(Multiple choices)*   1=TV 2=Radio 3=Cell phone 4=internet 5=Newspaper 6=Flyer 6=Visit to your home by a technician 7=Loudspeakers 8=I don't know |
|  |

*Mark the correct answer*

**Part C: Willingness of the community to receive the insecticide coating**

| 1. **Would you like to apply freely a transparent coating as a control method in your laundry tank?** |
| --- |
| 1 = Yes 2=No 3= I don’t know |

Thanks for your participation

*(Spanish version)*

*Estamos evaluando una nueva estrategia contra el dengue. Si me permite le realizaré unas preguntas cortas que no le quitarán mucho tiempo. Si usted se siente incómodo con alguna pregunta, puede no contestar o terminar la entrevista cuando lo desee.*

Código casa: _________________________ Código conglomerado: ____

1. **¿Podría darnos los siguientes detalles sobre las personas que viven en la casa, incluyéndose en la siguiente tabla?**

**SECCIÓN A: Características Sociodemográficas del hogar**

| Persona | Edad | Sexo  1=F  2=M  3=O | Educación  Primaria=1 Bachillerato= 2 Universidad= 3 No= 4 | ¿En este año ud.  o alguien de su hogar tuvo DZC?  1=Dengue 2=Zika 3=Chikunguna 4=No | ¿En qué Mes? | ¿Asistió al médico o unidad de salud?  1=SI 2=No | ¿Durante el día, donde permanece la mayor parte del tiempo (7 am -5 pm)? 1=En mi hogar o barrio, 2=Fuera del barrio |
| --- | --- | --- | --- | --- | --- | --- | --- |
| **1** |  |  |  |  |  |  |  |
| 2 |  |  |  |  |  |  |  |
| 3 |  |  |  |  |  |  |  |
| 4 |  |  |  |  |  |  |  |
| 5 |  |  |  |  |  |  |  |
| 6 |  |  |  |  |  |  |  |
| 7 |  |  |  |  |  |  |  |
| 8 |  |  |  |  |  |  |  |
| 9 |  |  |  |  |  |  |  |
| 10 |  |  |  |  |  |  |  |
| 11 |  |  |  |  |  |  |  |
| 12 |  |  |  |  |  |  |  |

**SECCIÓN B: Prácticas sobre el dengue y uso del agua**

| 1. **¿Usted aplica algún tipo de control en su tanque de lavadero para prevenirse del dengue?** |
| --- |
| 1= Uso peces 2 = Adición de cloro 3= Uso tapa tanque 4=Lavado y cepillado 5= Ninguno 6=No sé |
|  |
| 1. **¿En este año el servicio de salud ha aplicado algún insecticida tipo polvo o pastilla en su tanque de lavadero?** |
| 1= Si 2= No 3= No sé   1. **¿Cuáles de los depósitos usa para el consumo de agua?**   1= Tanque bajo 2=Agua de la llave 3=Otros depósitos, especifique 4=Ninguno   1. **¿Sabe usted en dónde ponen los mosquitos sus huevos o dónde se crían?**   1=Agua limpia 2=Agua sucia (charco) 3= Monte o Pasto 4= No sé   1. **¿Con cuál de estos medios le gustaría recibir información sobre el dengue?**  *(opción multiple)*   1=TV 2=Radio 3=Celular 4=Internet 5=periódico 6=Volante 6=Visita a su casa por técnico 7=Perifoneo 8=No sé |
|  |

*Marque la respuesta correcta*

**SECCIÓN C: Disposición de la comunidad para recibir el recubrimiento insecticida**

| 1. **¿Le gustaría que pongamos una pintura transparente como método de control de forma gratuita en su tanque de lavadero?** |
| --- |
| 1 = Si 2=No 3=No sé |

Gracias por participar
