# Supplementary material for: Risk of dengue, Zika, and chikungunya transmission in the metropolitan area of Cucuta, Colombia: cross-sectional analysis, baseline for a cluster-randomised controlled trial of a novel vector tool for water containers
Source: BMC Public Health. 2023 May 30;23:1000. doi: 10.1186/s12889-023-15893-4 (PMC10227988; doi:10.1186/s12889-023-15893-4)
Supplement: Supplementary file 2 — Additional file 2. [file 12889_2023_15893_MOESM2_ESM.docx]

Entomological survey

Form for extraction of entomological data

**House code: ________ Cluster: _____ Inspector: _______________ Date: ___/____/_____**

**Building type:** House: _ Car store: _ Health unit: _ Church: _ Grocery store: _ School: _ Restaurant: _ other**:** _ Public space:__

| **#** | | ***Water tanks** | | | | | | **Other water containers** | | | | | | | | | | ***** **Details of each water tank** | | | | | **Entomological indices** | | **Measures** | | | |
| --- | --- | --- | --- | --- | --- | --- | --- | --- | --- | --- | --- | --- | --- | --- | --- | --- | --- | --- | --- | --- | --- | --- | --- | --- | --- | --- | --- | --- |
|  | | Tanque bajo | | | | Storage tanks | Elevated tanks | buckets | Cans / small containers / Jars | Flowerpots | Tires | Sumps / sewers | Bottles (with water) | Gasoline bottles (*Pimpinas*) | Natural breeding site | Other: | Other: | Volume capacity (litres) | Water type (1=Watertap, 2=Rain, 3=other) | Tank location  (1=Inside, 0=Outside) | Shading  (1=Yes, 2=partly, 3=No) | Covered tank  (1=Yes, 2=partly, 3=No) | Larvae | Pupae count | Length (centimeters) | Width (centimeters) | Height (centimeters) |  |
|  |  | Concrete tanks | Plastic tanks | Tiled tanks | Metallic tanks |  |  |  |  |  |  |  |  |  |  |  |  |  |  |  |  |  | Positive (+),  Negative (-) | Total number |  |  |  |  |
| **Each water tank has its row (Water tank such as bottles etc. can be put together)** | 1 |  |  |  |  |  |  |  |  |  |  |  |  |  |  |  |  |  |  |  |  |  |  |  |  |  |  |  |
|  | 2 |  |  |  |  |  |  |  |  |  |  |  |  |  |  |  |  |  |  |  |  |  |  |  |  |  |  |  |
|  | 3 |  |  |  |  |  |  |  |  |  |  |  |  |  |  |  |  |  |  |  |  |  |  |  |  |  |  |  |
|  | 4 |  |  |  |  |  |  |  |  |  |  |  |  |  |  |  |  |  |  |  |  |  |  |  |  |  |  |  |
|  | 5 |  |  |  |  |  |  |  |  |  |  |  |  |  |  |  |  |  |  |  |  |  |  |  |  |  |  |  |
|  | 6 |  |  |  |  |  |  |  |  |  |  |  |  |  |  |  |  |  |  |  |  |  |  |  |  |  |  |  |
|  | 7 |  |  |  |  |  |  |  |  |  |  |  |  |  |  |  |  |  |  |  |  |  |  |  |  |  |  |  |
|  | 8 |  |  |  |  |  |  |  |  |  |  |  |  |  |  |  |  |  |  |  |  |  |  |  |  |  |  |  |
|  | 9 |  |  |  |  |  |  |  |  |  |  |  |  |  |  |  |  |  |  |  |  |  |  |  |  |  |  |  |
|  | 10 |  |  |  |  |  |  |  |  |  |  |  |  |  |  |  |  |  |  |  |  |  |  |  |  |  |  |  |
|  | 11 |  |  |  |  |  |  |  |  |  |  |  |  |  |  |  |  |  |  |  |  |  |  |  |  |  |  |  |
|  | 12 |  |  |  |  |  |  |  |  |  |  |  |  |  |  |  |  |  |  |  |  |  |  |  |  |  |  |  |
|  | 13 |  |  |  |  |  |  |  |  |  |  |  |  |  |  |  |  |  |  |  |  |  |  |  |  |  |  |  |
|  | 15 |  |  |  |  |  |  |  |  |  |  |  |  |  |  |  |  |  |  |  |  |  |  |  |  |  |  |  |
|  | 16 |  |  |  |  |  |  |  |  |  |  |  |  |  |  |  |  |  |  |  |  |  |  |  |  |  |  |  |
|  | 17 |  |  |  |  |  |  |  |  |  |  |  |  |  |  |  |  |  |  |  |  |  |  |  |  |  |  |  |
|  | 18 |  |  |  |  |  |  |  |  |  |  |  |  |  |  |  |  |  |  |  |  |  |  |  |  |  |  |  |

Comment: __________________________________________________________________________________

*(Spanish version)*

**Código casa: ________ Conglomerado: _____ Inspector: _______________ Fecha: ___/____/_____**

**Tipo de edificación:** Casa: _ Taller: _ Unidad de salud: _ iglesia: _ Tienda: _ Colegio: _ Restaurante: _ otro**:** _ Espacio Público:__

| **No.** | | ***Tanques de agua** | | | | | | **Otros depósitos con agua** | | | | | | | | | | ***Detalles de cada tanque de agua** | | | | | **Índices entomológicos** | | **Medidas** | | | |
| --- | --- | --- | --- | --- | --- | --- | --- | --- | --- | --- | --- | --- | --- | --- | --- | --- | --- | --- | --- | --- | --- | --- | --- | --- | --- | --- | --- | --- |
| SITIOS | | Tanque bajo | | | | Tanque almacenamiento | Tanque elevado | baldes | Latas / Envases / Jarras | Floreros | Neumáticos (Con o sin agua) | Sumideros/alcantarillas | Botellas (Con agua) | Galones ( Pimpinas) | Criaderos naturales | Otro: | Otro: | Capacidad en volumen (litros) | Tipo de agua  (1=la llave, 2=Lluvia, 3=otro) | Ubicación del tanque  (1=Adentro, 0=Fuera) | Sombreado  (1=Sí, 2=parcialmente, 3=No) | Cubierto  (Si=1, parcial= 2, No=3) | Larva | Conteo de pupas | Longitud | Anchura | Altura |  |
|  |  | Cemento | Plástico | Baldosa | Toneles metálicos |  |  |  |  |  |  |  |  |  |  |  |  |  |  |  |  |  | Positivo (+),  Negativo (-) | Número total |  |  |  |  |
| **Cada tanque de agua tiene su fila (Depósito de agua como botellas etc.. pueden ser puestos juntos)** | 1 |  |  |  |  |  |  |  |  |  |  |  |  |  |  |  |  |  |  |  |  |  |  |  |  |  |  |  |
|  | 2 |  |  |  |  |  |  |  |  |  |  |  |  |  |  |  |  |  |  |  |  |  |  |  |  |  |  |  |
|  | 3 |  |  |  |  |  |  |  |  |  |  |  |  |  |  |  |  |  |  |  |  |  |  |  |  |  |  |  |
|  | 4 |  |  |  |  |  |  |  |  |  |  |  |  |  |  |  |  |  |  |  |  |  |  |  |  |  |  |  |
|  | 5 |  |  |  |  |  |  |  |  |  |  |  |  |  |  |  |  |  |  |  |  |  |  |  |  |  |  |  |
|  | 6 |  |  |  |  |  |  |  |  |  |  |  |  |  |  |  |  |  |  |  |  |  |  |  |  |  |  |  |
|  | 7 |  |  |  |  |  |  |  |  |  |  |  |  |  |  |  |  |  |  |  |  |  |  |  |  |  |  |  |
|  | 8 |  |  |  |  |  |  |  |  |  |  |  |  |  |  |  |  |  |  |  |  |  |  |  |  |  |  |  |
|  | 9 |  |  |  |  |  |  |  |  |  |  |  |  |  |  |  |  |  |  |  |  |  |  |  |  |  |  |  |
|  | 10 |  |  |  |  |  |  |  |  |  |  |  |  |  |  |  |  |  |  |  |  |  |  |  |  |  |  |  |
|  | 11 |  |  |  |  |  |  |  |  |  |  |  |  |  |  |  |  |  |  |  |  |  |  |  |  |  |  |  |
|  | 12 |  |  |  |  |  |  |  |  |  |  |  |  |  |  |  |  |  |  |  |  |  |  |  |  |  |  |  |
|  | 13 |  |  |  |  |  |  |  |  |  |  |  |  |  |  |  |  |  |  |  |  |  |  |  |  |  |  |  |
|  | 15 |  |  |  |  |  |  |  |  |  |  |  |  |  |  |  |  |  |  |  |  |  |  |  |  |  |  |  |
|  | 16 |  |  |  |  |  |  |  |  |  |  |  |  |  |  |  |  |  |  |  |  |  |  |  |  |  |  |  |
|  | 17 |  |  |  |  |  |  |  |  |  |  |  |  |  |  |  |  |  |  |  |  |  |  |  |  |  |  |  |
|  | 18 |  |  |  |  |  |  |  |  |  |  |  |  |  |  |  |  |  |  |  |  |  |  |  |  |  |  |  |

Observación: __________________________________________________________________________________
